# The validation of a commercial enzyme-linked immunosorbent assay and the effect of freeze-thaw cycles of serum on the stability of cortisol and testosterone concentrations in Aceh cattle

**DOI:** 10.12688/f1000research.19804.3

**Published:** 2020-03-06

**Authors:** Gholib Gholib, Sri Wahyuni, Muslim Akmal, Muhammad Hasan, Muhammad Agil, Bambang Purwantara

**Affiliations:** 1 Physiology Laboratory, Faculty of Veterinary Medicine, Universitas Syiah Kuala, Banda Aceh, Aceh, 23111, Indonesia; 2 Anatomy Laboratory, Faculty of Veterinary Medicine, Universitas Syiah Kuala, Banda Aceh, Aceh, 23111, Indonesia; 3 Histology Laboratory, Faculty of Veterinary Medicine, Universitas Syiah Kuala, Banda Aceh, Aceh, 23111, Indonesia; 4 Clinical Laboratory, Faculty of Veterinary Medicine, Universitas Syiah Kuala, Banda Aceh, Aceh, 23111, Indonesia; 5 Department of Veterinary Clinic Reproduction and Pathology, Bogor Agricultural University, Bogor, Jawa Barat, 16680, Indonesia

**Keywords:** aceh cattle, enzyme-linked immunosorbent assay, cortisol, testosterone, analytical validation, biological validation, hormone stability, repeated freezing and re-thawing

## Abstract

**Background: **To obtain accurate measurements of cortisol (C) and testosterone (T) in Aceh cattle, commercial enzyme-linked immunosorbent assay (ELISA) kits need to be carefully validated. Moreover, repeated freeze-thaw cycles during the storage of the samples may affect the stability of the hormones in the serum. Here, the reliability of C and T concentration measurements in the serum of Aceh cattle, was tested using commercial C and T ELISA kits designed to measure human C and T concentrations. Further, the effect of repeated freeze-thaw cycles on the stability of C and T concentrations in the serum was evaluated.

**Methods: **Commercial C (Cat. no. EIA-1887) and T (Cat. no. EIA-1559) ELISA kits from DRG Instruments GmbH were validated through an analytical validation test (i.e., parallelism, accuracy, and precision) and a biological validation test (for C: effect of transportation on the C secretion; for T: the concentrations of T between bulls and cows). To test the effects of freeze-thaw cycles, cattle serum was subjected to the following treatments: (i) remained frozen at -20 ^O^C (control group); (ii) exposed to freeze-thaw cycles for two, four, six, and eight times (test groups).

**Results: **Parallelism, accuracy, and precision tests showed that both  C and T ELISA kits adequately measured C and T in the serum of Aceh cattle. Concentrations of C post-transportation were significantly higher than pre-transportation (p<0.01). Concentrations of T in bulls were significantly higher than in cows (p<0.01). After four to eight freeze-thaw cycles, C concentrations were significantly lower compared to the control group (all p < 0.05). In contrast, T concentrations remained stable (all p>0.05).

**Conclusions: **Commercial C (EIA-1887) and T (EIA-1559) ELISA kits are reliable assays for measuring serum C and T, respectively, in Aceh cattle. Repeated freeze-thaw cycles significantly affected the stability of serum C, but did not for T.

## Introduction

Cortisol (C) and testosterone (T) are steroid hormones. Cortisol is produced by the adrenal cortex ^1^, while T is produced by the Leydig cells in the testes ^2^ and also secreted in the adrenal cortex and ovaries in the small amounts ^3^. Cortisol has a key role in physiological stress responses. Accordingly, this hormone is commonly used as an indicator of stress ^4^. On the other hand, T plays an important role in male reproduction physiology. Thus, T concentrations can be used to assess male gonadal function ^2^. Measuring C and T concentrations can be used to monitor stress and reproduction in order to support the breeding management, because high concentrations of C can suppress the T production ^2^.

Steroid hormones can be measured by an enzyme-linked immunosorbent assay (ELISA) technique using various samples (e.g., plasma or serum ^5^, urine ^6^, feces ^7, 8^, saliva ^9, 10^, and hair ^11^). This technique is now widely used, as it is simple, rapid, convenient, relatively inexpensive, requires a lower sample volume, and particularly, as it is free of radioisotope waste ^12, 13^. Moreover, the availability of commercial ELISA kits makes it easy to apply this technique. However, because many commercial ELISA kits are usually designed for humans, using them for animals must be done with great caution and measurements are only trustworthy after the validation of the assay ^14^.

In order to reliably measure C and T concentrations in cattle using a human commercial ELISA kit, the ELISA kit needs to be validated analytically, physiologically or biologically ^5, 15^. Analytical validation can be performed by examining the specificity (cross-reactions), sensitivity, precision, and parallelism (linearity) of the ELISA kit ^1, 5^. In addition, it is crucial to perform the biological validation of the assay to examine the ability of the assay to differentiate the variation of the hormone concentration based on the physiological conditions of the animals ^15^. The biological validation of T measurements can be achieved by comparing the concentrations of T from individuals of different age (juvenile versus adult), or sex (male versus female) classes ^5^. For C measurements, the comparison of C concentrations of the same animal before and after some known stressful events (eg., capture, translocation, transportation, and agonistic interactions) can be used as a biological validation procedure ^15^.

Another critical issue for hormone measurements is the repeated freezing and re-thawing of the samples (freeze-thaw cycles) during the storage prior to the analyses ^16^. Freezing the serum or plasma at -20°C or lower is an ideal storage method ^17^. However, power outages frequently occur, particularly in developing countries, and they can last for a few hours up to a day. Manuals of commercial ELISA kits always explicitly warn to avoid freeze-thaw cycles, because repeated freezing and re-thawing of the samples may affect the stability of the hormones in the serum or plasma, for example: insulin in rats ^18^; adrenocorticotropic hormone in humans ^19^; and sex hormone-binding globulin (SHBG), progesterone, estrone, estradiol, and dehydroepiandrosterone sulfate (DHEAS) in humans ^20, 21^. Conversely, several studies have reported no effect of repeated freeze-thaw cycles on the stability of hormone concentrations, such as progesterone in female dogs (bitches) ^22^ and DHEAS, C, dihydrotestosterone, T, estradiol, and progesterone in humans ^23, 24^. These different results suggest that the stability may depend on the number of cycles, duration of cycles, and temperature during the repeated freezing and rethawing, species-specific differences, as well as on the type of hormones measured.

In 2011, the Ministry of Agriculture of Indonesia declared Aceh cattle to be a native Indonesian genome resource. This type of cattle can adapt well to the tropical environment and is important for meat production. Increasing the Aceh cattle population is very important for the fulfillment of the protein requirements of the human population in the Aceh region. In this respect, the measurement of C and T can be used for informing husbandry management and breeding programs for this animal. In preparation for the first study to monitor reproduction and stress physiology of Aceh cattle, the reliability of commercial C and T ELISA kits designed for human serum/plasma was tested to measure C and T concentrations in the serum of Aceh cattle. The objectives of the present study were: First, conduct an analytical validation test to examine the reliability of commercial C (Cat. no. EIA-1887) and T (Cat. no. EIA-1559) ELISA kits that accurately measure C and T concentrations, respectively in Aceh cattle; Second, perform a biological validation test to examine the reliability of commercial C ELISA kit (Cat. no. EIA-1887) to the response a stress event by comparing C concentrations before and after transportation and the reliability of T ELISA kit (Cat. no. EIA-1559) in discriminating the T concentrations from different sexes (bulls versus cows). Finally, the third objective of the study was to evaluate the stability of C and T concentrations in the serum of Aceh cattle after exposure to several repeated freeze-thaw cycles. The hypotheses are that 1) commercial C ELISA kit (Cat. no. EIA-1887) and T ELISA kit (Cat. no. EIA-1559) are reliable assays to measure C and T concentrations in the serum of Aceh cattle; 2) concentration of C should be higher following transportation and the T concentration should be higher in bulls; and 3) repeated freeze-thaw cycles affect the stability of C and T concentrations in the serum of Aceh cattle.

## Methods

### Ethical statement

The Institutional Committee of Animal Ethics of the Faculty of Veterinary Medicine, Universitas Syiah Kuala approved the use of all experimental animals in this study (Ref: 33/KEPH/VI/2019) ^25^ (see *Extended data*). All efforts were made to ameliorate harm to the animals, such as: cattle were placed in the clamp cage to ensure quick, easy and safe collection of the blood sample causing minimal distress; blood samples were collected via the jugular vein without sedation, causing minimal distress. During the study, a member of the study team, accompanied by a veterinarian, observed their behavior for signs of excessive distress or sickness. None of the procedures performed in this study resulted in distress, sickness behavior or weight loss.

### Study animals

Samples were collected from 32 adult Aceh cattle consisting of 16 adult males (bulls), and 16 adult females (cows). The age of the cattle ranged from two to five years old, and weighing 150–300 kg. Samples were collected during two weeks in April 2019 and three weeks in November 2019. For the biological validation test of T ELISA kit, all cattle (16 bulls and 16 cows) were used, whereas for the biological validation of C ELISA kit, 16 cattle (8 bulls and 8 cows) were used. This sample size was calculated based on Federer’s formula: (t-1)(n-1) ≥ 15, where t is the number of treatments and n is the number of animals. The sample size calculated using this formula is 16. Sixteen bulls and 10 cows were sampled from a smallholder farmer at Darussalam, Aceh Besar, Aceh Province. These cattle were housed in stables, with each cattle was separated by a wooden partition (individual housing system). Six cows were sampled from a teaching farm (UPT hewan coba), Faculty of Veterinary Medicine, Universitas Syiah Kuala, Banda Aceh. These cows were housed together in stables (colony housing system). Cattle housing was equipped with feed and water troughs. Feed and water were consumed by the cattle *ad libitum*. Feed given to the cattle is grass at 10% of body weight and 1% of body weight of rice bran and tofu dregs.

### Collection and processing of blood samples

Blood samples (around 5 ml per animal) were collected by a veterinarian from the jugular vein using standard operating procedures without sedation. For the biological validation of C, blood samples were collected in the afternoon (16.00 to 18.00), four hours after transportation, while for the biological validation of T, blood samples were collected in the morning (06.00 to 08.00). Afterward, the blood was allowed to clot at room temperature for between 30 minutes to 2 hours. Then, the serum was separated from the red blood cells by centrifugation at 1200xg for 10 minutes at 4°C. After that, the supernatant (serum) was immediately transferred to polypropylene tubes (Eppendorf Safe-Lock tubes) and stored at -20°C until hormone measurements.

### Validation of commercial C and T ELISA kits

To test the capability of the commercial C and T ELISA kits in quantifying concentrations of T and C in the serum of Aceh cattle, the commercial C (Cat. no. EIA-1887) and T (Cat. no. EIA-1559) ELISA kits produced by DRG Instruments GmbH, Germany, were evaluated using analytical and biological validations. The procedure for assay validation was conducted as described by Rangel-Negrin *et al.* ^15^ and Gholib *et al.* ^5^. Briefly, the analytical validation was comprised of parallelism (i.e. running serial dilution of Aceh cattle serum 1:2 to 1:16, assayed together with C and T standards, and comparing the slope of expected dose versus percent bound of diluted Aceh cattle serum with the slope of C and T standards), accuracy (i.e. adding known quantities of hormone to C and T standards and calculating the percentage of recovery), and precision (i.e., measuring some low-quality controls (QC L) and high-quality controls (QC H) in one microplate to calculate intra-assay coefficients of variation (CV) and some QC L and QC H in several microplates to calculate inter-assay CV. The sensitivity was reported as provided by the manufacturer.

For the biological validation of C, we examined the effects of transportation on the C secretion. We used 16 of the Aceh cattle described above (eight bulls and eight cows). Bulls and cows were transported using different road vehicles (open car) around Banda Aceh at 11.00 for an hour (~40 km/hour). During transportation, cattle were secured using a rope around their neck and bulls and cows were transported using different vehicles. For pre-transportation samples, blood samples were collected a day before transporting the cattle at 06.00 to 08.00. For post-transportation samples, blood samples were collected four hours after transportation at 16.00 to 18.00. Blood samples were then processed as described above. For the biological validation of T, we compared the concentrations of T from 16 bulls and 16 cows. For this purpose, blood sample was collected once for each cattle in the morning at 06.00 to 08.00.

### Experiment design of freeze-thaw cycles

To evaluate the stability of C and T concentrations in serum exposed to repeated freeze-thaw cycles, we took seven serum samples (three bulls and four cows) collected from the previous experiment (biological validation of C and T) to be used for the freeze-thaw cycle experiment, in order to avoid several blood sample collections. Each serum sample was then divided into five aliquots and transferred into 1.5 ml micro-tubes (Eppendorf Safe-Lock tubes; total 35 aliquots; 0.2 ml per tube), closed tightly and sealed with parafilm. All aliquots were subsequently stored frozen at -20°C. Later, those aliquots were subjected to the following treatments: (i) aliquots remained frozen at -20°C until the time of hormone analysis as a control group (N=7); and (ii) aliquots were exposed to repeated freeze-thaw cycles for two, four, six, and eight cycles as test groups (N=7 for each group). The serum was thawed for six hours by placing the tube in a room without an air conditioner (mean ± standard deviation of temperature 27.6±0.7°C). Afterward, the serum was refrozen for 24 hours prior to re-thawing. After all freeze-thaw cycles were completed, C and T concentrations were measured for all serum samples together.

### C and T concentration measurements

The concentration of C was measured using a commercial C ELISA kit (Cat. no. EIA-1887, DRG Instruments GmbH, Germany). The assay utilizes a monoclonal anti-cortisol antibody and C labeled with horseradish peroxidase as an enzyme conjugate. The measurement of C was conducted following the instructions of the manufacturer ( DRG diagnostics). In brief, 20 μl of each standard, control, and samples (serum) in duplicate were dispensed with new disposable tips into appropriate wells on a microplate coated with C monoclonal antibody. After that, each microplate well was filled with 200 μl enzyme conjugate, thoroughly mixed, and then incubated for 60 minutes at room temperature. After incubation, the solution in the wells was briskly shaken out and rinsed out with 350 μl diluted washing solution per well four times. Furthermore, 100 μl substrate solution (tetramethylbenzidine) was added to each well of the microplate, which was then re-incubated for 15–20 minutes at room temperature. After that, the enzymatic reaction was stopped by adding 100 μl of stop solution (0.5 M H _2_SO _4_) to each well. Finally, absorbance was determined by using an ELISA reader (xMark™ Microplate Absorbance Spectrophotometer, Bio-Rad Laboratories Inc.) at 450 nm. The C concentration was then calculated using the Microplate Manager ^®^ 6 Software (Bio-Rad Laboratories Inc.).

The concentration of T was measured using a commercial T ELISA kit (Cat. no. EIA-1559, DRG Instruments GmbH, Germany). The assay utilizes a mouse monoclonal anti-testosterone antibody and T labeled with horseradish peroxidase as an enzyme conjugate. This assay has been previously validated successfully for measuring T concentrations in Kacang goats ^5^. Testosterone measurements were conducted following the instructions of the manufacturer ( DRG diagnostics) and as described by Gholib *et al.* ^5^. In brief, 25 μl of each standard, control, and samples (serum) in duplicate were dispensed with new disposable tips into appropriate wells on a microplate coated with T monoclonal antibody. After that, each microplate well was filled with 200 μl enzyme conjugate, thoroughly mixed, and then incubated for 60 minutes at room temperature. Following this, the microplates were treated and absorbance was measured as described above for C.

### Data analysis

Normality distribution of the data was tested using the Shapiro-Wilk test prior to statistical analysis. For the analytical validation, the parallelism between serial dilutions of two selected serum samples and standard curves was determined by a test of equality of two slopes using Logarithmic Regression ^26^. For the biological validation, C data before and after transportation and T data of bulls and cows showed a normal distribution (p>0.05). A paired t-test was used for C, whereas for T, an independent t-test was used. For the freeze-thaw cycles experiments, the proportion of change in C and T concentrations relative to the control was calculated as (a _n_– x _n_/x _n_) x 100, where a _n_ is the nth sample value in each freeze-thaw cycles series (two, four, six, eight times) and x _n_ is the value at time zero (control) of the nth sample ^8, 16^. The C data were normally distributed (p>0.05), whereas the T data was not (p<0.05). Therefore, for C, a one-way repeated measures ANOVA followed by *post hoc* analysis using the Bonferroni test was conducted, whereas a Friedman repeated-measures ANOVA on ranks was set up to analyze T concentrations ^8^. We used SigmaPlot 11.0 to create graphs and IBM SPSS 20 to carry out the statistical analysis. All statistical tests were two-tailed and the significance level was set at 0.05.

## Results

### The validity of commercial ELISA kits for measuring C and T in the serum of cattle

**Figure 1.**
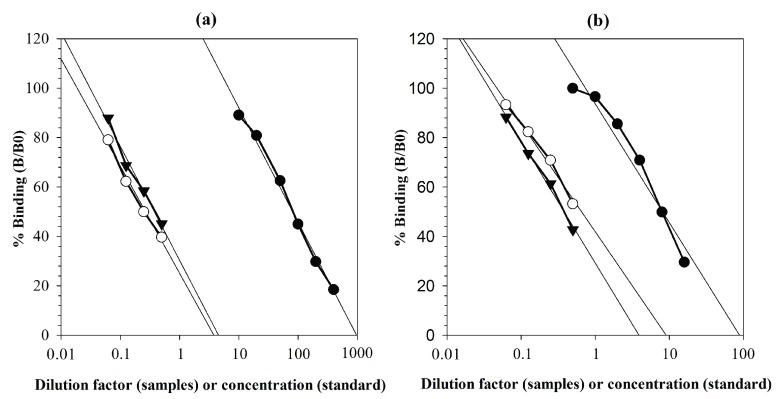
Curves of parallelism test from the serial dilution of tested samples (serum of Aceh cattle) and cortisol and testosterone standards are presented. **a**) Cortisol (C) enzyme-linked immunosorbent assay (ELISA) kit: serum of post-transportation / sample one (white circle) and serum of pre-transportation / sample two (black triangle down) were diluted 1:2 to 1:16 in assay buffer and measured with serial C standards (black circle) ranging from 10 to 400 ng/ml. The curve of sample one, sample two, and C standards produced almost identical *R* ^2^ values: 0.987, 0.945, and 0.992, respectively. **b**) Testosterone (T) ELISA kit: serum of bull 1 / sample one (white circle) and serum of bull 2 / sample two (black triangle down) were diluted 1:2 to 1:16 in assay buffer and measured with serial T standards (black circle) ranging from 0.5 to 16 ng/ml. The curve of sample one, sample two, and T standards produced almost identical *R* ^2^ values: 0.964, 0.993, and 0.945, respectively.

The serial dilution of the selected serum (for C: serum of post-transportation / sample one, and serum of pre-transportation /sample two; for T: serum of bull 1 / sample one, and serum of bull 2 / sample two) showed displacement curves that were parallel to C and T standard curves ( Figure 1). From dose-response curve, the value of slope on C standard, sample one, and sample two were -20.106, -18.827, and -19.433, respectively. The test of equality of two slopes showed that the slope of C standard was parallel or not significantly different with the slope of sample one (t = -0.218, p = 0.462) and slope of sample two (t = -0.674, p = 0.443; Table 1). Similar results were also obtained for T. Slope of T standard (b = -21.584) was parallel or not significantly different with slope of sample one (b = -17.373, t = -0.067, p = 0.332) and slope of sample two (b = -21.480, t = 0.376, p = 0.212; Table 1). The parallelism, dose-response, accuracy, and precision (coefficients of variation/CV of intra-and inter-assay) are presented in Table 1.

**Table 1.** Results of the analytical validation of the commercial cortisol (DRG, Cat. No. EIA-1887) and testosterone (DRG, Cat. No. EIA-1559) enzyme-linked immunosorbent assay (ELISA) kits.

Measured parameters	Cortisol ELISA kit	Testosterone ELISA kit
Parallelism		
- Standard with sample one	Parallel, t = -0.218, p = 0.462	Parallel, t = -0.067, p = 0.332
- Standard with sample two	Parallel, t = -0.674, p = 0.443	Parallel, t = 0.376, p=0.212
Dose response curve		
- Sample one	y = -18.827 Ln (x) + 25.050 R ^2^ = 0.987, p = 0.006	y = -17.373 Ln (x) + 43.792 R ^2^ = 0.964 , p = 0.018
- Sample two	y = -19.433 Ln (x) + 30.350 R ^2^ = 0.945, p = 0.028	y = -21,480 Ln (x) + 29.218 R ^2^ = 0.993, p = 0.003
- Standard	y = -20.106 Ln (x) + 138.345 R ^2^ = 0.992, p = 0.005	y = -21,584 Ln (x) + 95.239 R ^2^ = 0.945, p = 0.004
Accuracy±SD (%) (N=6)	104.89 ± 7.09	103.37 ± 9.62
Coefficient variation (CV) of intra-assay (%)		
- Low-quality control (N=6)	8.40	7.32
- High-quality control (N=6)	4.86	6.62
Coefficient variation (CV) of inter-assay (%)		
- Low-quality control (N=6)	12.45	10.70
- High-quality control (N=6)	6.80	8.91
Sensitivity (ng/ml) ^a^	2.5	0.083

^a^ = value in the manual protocol of assay from manufacturer

**Figure 2.**
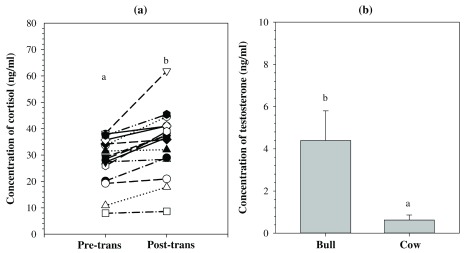
Results of the biological validation of the commercial enzyme-linked immunosorbent assay (ELISA) kits. **a**) Concentrations of cortisol (C) before (pre-trans) and after (post-trans) transportation measured using a commercial C ELISA kit (DRG, Cat. No. EIA-1887). **b**) Concentrations of testosterone (T) in bulls and cows measured using a commercial T ELISA kit (DRG, Cat. No. EIA-1559). Different superscripts above line symbols and histogram indicate a significant difference between groups (p < 0.05).

The mean (± SD) of the C concentrations in the samples post-transportation (34.81 ± 12.38 ng/ml) was significantly higher compared to pre-transportation samples (27.83 ± 9.22 ng/ml; t = -4.670, p = 0.000; Figure 2A). The mean (± SD) of the T concentrations in bulls (4.39 ± 1.41 ng/ml) was significantly higher than the T concentrations in cows (0.63 ± 0.24 ng/ml; t = 10.552, p = 0.000; Figure 2B). Results on the analytical and biological validations indicated that both C and T ELISA kits were reliable assays for measuring C and T concentrations in serum of Aceh cattle. Raw values of analytical validation results are given in Dataset 1 ^27^, whereas raw values of biological validation results are given in Dataset 2 v2 ^28^ (see *Underlying data*).

### C and T stabilities after exposure to repeated freeze-thaw cycles

**Figure 3.**
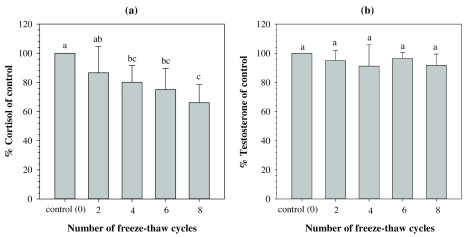
Percentages of hormone concentrations in the serum exposed to repeated freeze-thaw cycles compared to control. **a**) Cortisol (C). **b**) Testosterone (T). Values represent mean±SD relative to control (100%). Different superscripts above histogram indicate a significant difference between groups (p < 0.05).

Repeated freeze-thaw cycles in serum significantly affected the stability of C concentrations (F[4,30] = 11.681, p<0.001, N=7). *Post hoc* analysis showed that after four to eight freeze-thaw cycles, the C concentrations were significantly lower than those of the control group (all p < 0.05; Figure 3A). In contrast to the C concentrations, the T concentrations remained stable after exposure to two to eight freeze-thaw cycles (χ ^2^[4] = 7.626, p = 0.106, N = 7; Figure 3B). The mean percentage of change in C and T concentrations ranged between 13.43 to 33.94% and 3.55 to 8.33%, respectively, relative to the control group ( Figure 3). Raw values of C and T concentrations are given in Dataset 3 ^29^ (see *Underlying data*).

## Discussion

The current study demonstrates that C and T concentrations in the serum of Aceh cattle can be accurately measured using the commercial C and T ELISA kits designed for human C and T measurements. The results of the parallelism test show that the slope of the diluted sample curves was parallel to the standard curves of commercial C and T ELISA kits. In addition, concentrations of C and T decreased significantly following the dilution levels. Furthermore, these assays also show high accuracy (~100%) and precision (CV < 10% and < 15% for intra- and inter-assay, respectively). Therefore, both the commercial C ELISA kit and the commercial T ELISA kit tested can be used to reliably measure C and T concentrations in the serum of Aceh cattle.

A significant increase in C secretion after transportation was detected by the commercial C ELISA kit tested. This result was predicted because C is the main modulator of physiological stress and it usually increases in response to a stressor ^30^. The commercial T ELISA kit also appeared reliable in its ability to discriminate T concentrations between bulls and cows. Testosterone concentrations in bulls were more than five times higher compared to cows. This result was expected because T is the major androgen produced by the Leydig cells of the testes ^31^, whereas only small amounts are secreted in the adrenal cortex and ovaries of females ^3^.

Our results indicated that the biological validation of commercial C and T ELISA kits can be performed using C measurements in relation to a stressful event and T measurements of different sexes. It is crucial to conduct such as validation to ensure the biological meaningfulness of the analyses ^32, 33^. A commercial C ELISA kit (DRG, Cat. No. EIA-1887) and a commercial T ELISA kit (DRG, Cat. No. EIA-1559) are commercial ELISA kits developed for measuring hormone concentrations, particularly in human serum/plasma. These assays use an antibody highly specific to either C or T. The antigen (C or T) in the serum of Aceh cattle can bind correctly with the antibody (anti-cortisol and anti-testosterone) in these ELISA kits ^5^. This is because the side-chains of the carbon compounds of C (C-21) ^34^ and T(C-19) ^3^ have similar structures in vertebrates and the basic structure of steroid hormones is derived from the cyclopentanoperhydrophenanthrene structure. The validation of commercial ELISA kits for measuring hormones in animals has been successfully conducted in several species, such as progesterone in serum of cattle using human progesterone ELISA kit (Clinpro International Co. LLC, Union City, CA 94587, USA) ^35^, estradiol-17-beta and inhibin A in plasma of buffalo using human ELISA kit (For estradiol: Cat No. EIA-2693, DRG Instruments GmbH, Marburg, Germany; For Inhibin A: Inhibin A DSL-10-28100, Diagnostic System Laboratories Inc, Webster, Texas, USA) ^36^, C in plasma of horses using Immunotech kit ^37^, and T in serum of Kacang goats using T ELISA kit (Cat No. EIA-1559, DRG Instruments GmbH, Marburg, Germany) developed for human ^5^.

In addition to the validation of ELISA kits, the effects of repeated freeze-thaw cycles on the stability of C and T concentrations in serum were tested. Concentrations of C decreased significantly after the exposure to four, six, and eight freeze-thaw cycles, while T concentrations did not. Concentrations of C declined up to 33.94% after eight freeze-thaw cycles, whereas the percentage changes in T concentrations were less than 10% in all test groups. These findings show that T is more resilient to freeze-thaw cycles compared to C. The reason why C concentrations decreased after exposure to repeated freeze-thaw cycles and T concentrations did not, it is not entirely clear. For C, it is possible, however, that the serum left at high temperatures (27.6±0.7°C) during the six hours of the thawing process might facilitate the increased degradation of C ^16^. Consequently, less antigen in the serum binds with the antibody of the assay ^38^. Thus, C concentrations in all test groups were lower than the control group.

From a practical point of view, our results suggest that commercial C (EIA-1887) and T (EIA-1559) ELISA kits can accurately measure C and T concentrations, respectively, in the serum of animals such as Aceh cattle. It can be advantageous to use these commercial assays over assays specifically designed for animals because of their relatively low price. On the other hand, repeated freeze-thaw cycles must be considered, especially for the C measurement. Therefore, conditions that may potentially cause repeated freeze-thaw cycles of samples designated for hormone analysis (e.g. frequent power outages) should be avoided. Some efforts can be performed to prevent the possibility of repeated freeze-thaw cycles ^16^. First, installing a backup generator to supply electricity when there are power outages. Second, filling each freezer with a blanket of ice packs to maintain the temperature inside the freezer during the power outage. Third, averting the use of an aliquot of serum/plasma for several analyses at different times. Fourth, dividing serum into several aliquots when several analyses are performed from the same sample.

This study has some limitations particularly information regarding the substantive reason for decreasing C concentrations after repeated freeze-thaw cycles which is still unclear. To elucidate this reason, the biochemistry of the serum after exposure to freeze thaw-cycles needs to be investigated in future studies. Moreover, it is unclear whether these assays are also reliable for other species, so they would need a validation test for each species. Apart from the fact that there are limitations, the results are important to support further studies in Aceh cattle, particularly for maintaining and growing the Aceh cattle population to ensure food security in the Aceh region and Indonesia as a whole.

In conclusion, our study shows that based on the analytical and biological validation tests, commercial C (EIA-1887) and T (EIA-1559) ELISA kits are reliable assays for measuring C and T, in serum of Aceh cattle. Moreover, biological validation showed that fluctuations of C and T in serum of Aceh cattle have a clear biological significance reflecting on the physiological condition of Aceh cattle. Results of the study also demonstrate that more than two repeated freeze-thaw cycles significantly affected the stability of serum C concentrations, but up to eight repeated freeze-thaw cycles did not significantly affect the stability of serum T concentrations.

## Data availability

### Underlying data

Figshare: Dataset 1. https://doi.org/10.6084/m9.figshare.8342504.v2 ^27^

This project contains the following underlying data:

- Analytical validation data.csv (raw values of analytical validation results included data of parallelism, accuracy, and precision [coefficients of variation/CV of intra-and inter-assay])

Figshare: Dataset 2 v2. https://doi.org/10.6084/m9.figshare.11678352 ^28^

This project contains the following underlying data:

- Data of Biological validation.csv (raw values of biological validation results: cortisol concentrations before and after transportation and testosterone concentrations in bulls and cows of Aceh cattle)

Figshare: Dataset 3. https://doi.org/10.6084/m9.figshare.8342720.v1 ^29^

This project contains the following underlying data:

- Data of freeze-thaw cycles on cortisol and testosterone.csv (raw value of cortisol and testosterone concentration in Aceh cattle serum after exposure repeated freeze-thaw cycles)

Data are available under the terms of the Creative Commons Attribution 4.0 International license (CC-BY 4.0).

### Extended data

Figshare: Extended data 1. https://doi.org/10.6084/m9.figshare.8487830.v1 ^25^

This project contains the following extended data:

- Ethical Clearance Approval Gholib *et al.*.pdf (certificate of ethical clearance approval for using animals)

Data are available under the terms of the Creative Commons Zero "No rights reserved" data waiver (CC0 1.0 Public domain dedication).
