# Supplementary material for: The validation of a commercial enzyme-linked immunosorbent assay and the effect of freeze-thaw cycles of serum on the stability of cortisol and testosterone concentrations in Aceh cattle
Source: F1000Res. 2020 Mar 6;8:1220. Originally published 2019 Jul 30. [Version 3] doi: 10.12688/f1000research.19804.3 (PMC7047911; doi:10.12688/f1000research.19804.3)
Supplement: Peer review history

# Peer review: Reviewer response for version 2

**DOI:** 10.5256/f1000research.24459.r59513

**Published:** 2020-02-27

**Authors:** Annaïs Carbajal

**Affiliations:** 1 Department of Animal Health and Anatomy, Autonomous University of Barcelona, Barcelona, Spain

The authors have adequately addressed all the issues suggested by the reviewers and therefore the manuscript has greatly improved.

 There are a few minor changes that you will find in the document attached here.

 I also suggest that the authors have their manuscript checked by an English speaker in order to further improve the ms.

Is the work clearly and accurately presented and does it cite the current literature?

Yes

If applicable, is the statistical analysis and its interpretation appropriate?

Partly

Are all the source data underlying the results available to ensure full reproducibility?

Yes

Is the study design appropriate and is the work technically sound?

Partly

Are the conclusions drawn adequately supported by the results?

Partly

Are sufficient details of methods and analysis provided to allow replication by others?

Yes

Reviewer Expertise:

Endocrinology and animal stress

I confirm that I have read this submission and believe that I have an appropriate level of expertise to confirm that it is of an acceptable scientific standard.

# Response

**Published:** 2020-03-03

**Authors:** Gholib Gholib

**Affiliations:** Universitas Syiah Kuala, Indonesia

**Response to Reviewer 1 and 2.**

First, we would like to thanks to reviewer 1 and 2 for their review and approved our paper (version 2). Minor comments and suggestions from reviewer 2 have helped us to further improve this paper. We have changed the paper according to all points raised. Each objective has been added with the hypothesis. Explanations of sample one and sample two used for parallelism test were added. Some grammatical errors and typing errors have been improved.

# Peer review: Reviewer response for version 2

**DOI:** 10.5256/f1000research.24459.r59514

**Published:** 2020-02-05

**Authors:** Pudji Astuti

**Affiliations:** 1 Department of Veterinary Medicine, Gadjah Mada University (UGM), Yogyakarta, Indonesia

Is the work clearly and accurately presented and does it cite the current literature?

Yes

If applicable, is the statistical analysis and its interpretation appropriate?

Yes

Are all the source data underlying the results available to ensure full reproducibility?

Yes

Is the study design appropriate and is the work technically sound?

Yes

Are the conclusions drawn adequately supported by the results?

Yes

Are sufficient details of methods and analysis provided to allow replication by others?

Yes

Reviewer Expertise:

NA

I confirm that I have read this submission and believe that I have an appropriate level of expertise to confirm that it is of an acceptable scientific standard.

# Peer review: Reviewer response for version 1

**DOI:** 10.5256/f1000research.21725.r54435

**Published:** 2019-10-16

**Authors:** Annaïs Carbajal

**Affiliations:** 1 Department of Animal Health and Anatomy, Autonomous University of Barcelona, Barcelona, Spain

The authors validated an EIA for testosterone and cortisol detection in serum of Aceh cattle. They also performed the biological validation of both hormones in serum. And finally, they evaluated the effect of freeze-thaw cycles on the stability of these hormones in serum. 

 Although the technique they present is not new, the validation the authors perform are imperative for the proper hormone interpretation in every matrix, and thus this should be done for each species, matrix and assay of interest. The study has a robust experimental design despite using a low number of samples in some experiments. Results they present are very useful and may provide guidance for future studies to perform appropriate validations of their methods.

 My major concern is why the authors discuss that T is affected by the freeze-thaw cycles if results do not indicate the same. Additionally, I would suggest dividing the aims in three: analytical validation, biological validation and freeze-thaw cycles, as they may provide three different results and conclusions. I have also added some minor comments which can be found in the pdf document that I hope they will be useful for the authors.

Is the work clearly and accurately presented and does it cite the current literature?

Yes

If applicable, is the statistical analysis and its interpretation appropriate?

Partly

Are all the source data underlying the results available to ensure full reproducibility?

Yes

Is the study design appropriate and is the work technically sound?

Partly

Are the conclusions drawn adequately supported by the results?

Partly

Are sufficient details of methods and analysis provided to allow replication by others?

Yes

Reviewer Expertise:

Endocrinology and animal stress

I confirm that I have read this submission and believe that I have an appropriate level of expertise to confirm that it is of an acceptable scientific standard, however I have significant reservations, as outlined above.

# Response

**Published:** 2020-01-23

**Authors:** Gholib Gholib

**Affiliations:** Universitas Syiah Kuala, Indonesia

Response to Reviewer 2.

First, we would like to thanks to the reviewer 2 for reviewing our paper and gave approved with reservations . Her comments and suggestions have helped us to further improve the paper. As you will see in the response-to-reviewer  below, we have answered all points raised and have changed the paper accordingly. We very much hope that the paper is now finally approved.

1. New sentence should not start with an abbreviation. This needs to be rectified in the whole text

***Response: All abbreviation in new sentences have been changed throughout text.***

2. T is also produced by the ovaries and the adrenals

**Response: Yes, you are right,** **T is also produced by the ovaries and the adrenals.** **An explanation has been added in revised paper**

3. Could also be due to species-specific differences? **Response: Yes, it can be influence by species-specific differences, Thank you very much for the revision. It has been added to the revised paper.**

4. The aims of the study are not fully clear here. As three different results are discused, I would recommend dividing the aims in 3 clear objectives: Analytical validation, biological validation and freeze-thaw cycles

**Response: Thank you very much for your suggestion. Now, The aims were dividedinto three objectives. The objective of the present study were: First, conducting an analytical validation test to examine the reliability of commercial C (Cat. no. EIA-1887) and T (Cat. no. EIA-1559) ELISA kits that accurately measure C and T concentrations, respectively in Aceh cattle; Second, performing a biological validation test to examine the reliability of commercial C ELISA kit (Cat. no. EIA-1887) to the response a stress event by comparing C concentrations before and after transportation and the reliability of T ELISA kit (Cat. no. EIA-1559) ELISA kits in discriminating the T concentrations from different sexes (bulls versus cows). The hypothesis is that C concentrations should be higher following transportation and the T concentration should be higher in bulls. Finally, the stability of C and T concentrations in the serum of Aceh cattle after exposure to several repeated freeze-thaw cycles was evaluated. For details, please see in the revised paper.**

5. This is not totally true since only 5 individuals are used for the C validation.

**Response: Thank you for your correction. Thank you for your correction. *We have collected additional samples ( 10 bulls and 10 cows) to increase the number of samples. Now, in total 32 Aceh cattle were used:  16 bulls and 16 cows. *For the *testosterone validation test, all cattle ( 16 bulls and 16 cows) were used. For cortisol validation test,  16 cattle ( 8 bulls and 8 cows ) were used which is taken from 32 Aceh cattle above.  The explanation  has been added in the revised paper.***

6. Information is lacking here. For example, why did you collect samples at 16h and 6h? This information should go to the subsection  "biological validation" - experimental design.

**Response: For biological validation of C, Samples were collected at 16 h, four hours after the trip to monitor the increase in Cortisol after the transportation. For biological validation of T, Samples were collected in the morning (6h-8h) because in some speceies including cattle, there is diurnal variation of T in blood which concentration of T ishigher in the morning compared to afternoon. This explanation has been added to the subsection of biological validation.**

 7. Hypothesis are written in the introduction

**Response: Hypothesis has been deleted in this section and moved to the subsection “ introduction”.**

8. Why serum was collected a day before and not immediately before transport? Please, specify the time of day when samples were collected. **Response: Sample was collected a day before to prevent the stress in cattle and sometimes blood collection in cattle needs some times. More explanation has been added in the revised paper**

9.This is mean and standard deviation? Please, specify

**Response: Thank you very much for your correction, it has been added in the revise paper.**

10. Ref 24 extended data 1 shows: Certificate of ethical clearance approval for using animals for study on monitoring reproduction and stress in aceh cattleData on the test of equality is not provided. Here the reference should be ref 26.

Indicate the name of the statistical model used to "test of equality of two slopes"

**Response: Model used for the test of equality of two slopes is Logarithmic regression. Thank you for your correction, the reference No. was also changed.**

11. More information is needed to interpret the data.Parallelism: indicate which test was made in order to obtain results of t and p

Dose response curve: is this a different test or they are results from the parallelism test? Do the equations provide extra information with regard to the parallelism test results (t and p)?

**Response: Thank you for your suggestion. For the parallelism, a test of equality of two slopes was used to compare between standard slope (C and T) and slope of sample one and sample two obtained from the regression equations. From the equations {dose respone curve) containing value of slope for standard , sample one and two. Based on the results, we compared the standard slope (C and T) with the slope of sample one and sample to see whether they are parallel to standard slope based on the the pvalueand t value. Since the pValue >0.05, it is indicated that these slopes are similar/parallelwith the standard slope (not significant different). More explanation has been added in the revised paper**

12. Table 2 and Figure 3 provide the same information. If percentages were used to perform statistics, I suggest removing Table 2.

**Response: Thank you for your suggestion, the Table 2 has been deleted fronm the revised paper**

13. Reviewer 2: used to reliably measure C and T concentrations in serum of Aceh cattle **Response: Thank you, the specific assay used in each study has been added in the revised paper.**

**14. **Reviewer 2: Please specify the matrix you are talking about

**Response: Thank you, the specific assay used in each study has been added in the revised paper.**

**"The validation of commercial ELISA kits for measuring hormones in animals has been successfully conducted in several animals, such as progesterone in cattle using human progesterone ELISA kit (Clinpro International Co. LLC, Union City, CA 94587, USA) , estradiol-17-beta and inhibin A in buffalo using human ELISA kit (For estradiol: Cat No. EIA-2693, DRG Instruments GmbH, Marburg, Germany; For Inhibin A: Inhibin A DSL-10-28100, Diagnostic System Laboratories Inc, Webster, Texas, US), C in horses using Immunotech kit , and T in Kacang goats using T ELISA kit (Cat No. EIA-1559, DRG Instruments GmbH, Marburg, Germany) developed for human."**

15. Your results suggest the opposite regarding the T levels Your results suggest the opposite regarding the T levels

**Response: Thank you for your correction, Although, T wes decresed less 10%, but this is not significant. You are right, T is not significantly affected by freeze thaw cycles. The explanation has been improved**.

16. Consider shortening the sentence or dividing the information in 2-3 shorter sentences. This way, it will be easier for the reader to understand.

**Response: Thank you very much for your suggestion. The sentence has been divided into 4 shorter sentences.**

17. Reviewer 2: Same as before. If the T concentrations remained stable, why do you say that T concentrations decreased? **Response: Thank you, it has been corrected in the revised paper.**

18. Reviewer 2: An important conclusion is lacking: The biological validation suggests that fluctuations of C and T in serum of Aceh cattle have a clear biological significance.

**Response: Thank you for your suggestion. An additional  conclusion has been added in the revised paper.**

**"In conclusion, our study shows that based on the analytical and biological validation tests, commercial C (EIA-1887) and T (EIA-1559) ELISA kits are reliable assays for measuring  C and T,  in serum of Aceh cattle. Moreover, biological validation showed that fluctuations of C and T in serum of Aceh cattle have a clear biological significance reflecting to the physiological condition of Aceh cattle. Results of the study also demonstrate that more than two repeated freeze-thaw cycles significantly affected the stability of serum C concentrations, but up to eight repeated freeze-thaw cycles did not significantly affect the stability of serum T concentrations."**

# Peer review: Reviewer response for version 1

**DOI:** 10.5256/f1000research.21725.r52712

**Published:** 2019-09-03

**Authors:** Pudji Astuti

**Affiliations:** 1 Department of Veterinary Medicine, Gadjah Mada University (UGM), Yogyakarta, Indonesia

1. The details comment of the paper are attached (Please see attachment).
2. Overall the content are complete including the validation test and the Biological validation are conducted.
3. The theory of similarity base chemical formula namely cyclopentano perhydro phenanthrene both of human and animal should be added.
4. The information standard of parallelism should be added.
5. Although the number of samples is less than specified (based on the Frederer formula), the results can still be accounted for because the amount of 12 samples is still very sufficient considering the large number of replications.

Is the work clearly and accurately presented and does it cite the current literature?

Yes

If applicable, is the statistical analysis and its interpretation appropriate?

Yes

Are all the source data underlying the results available to ensure full reproducibility?

Yes

Is the study design appropriate and is the work technically sound?

Yes

Are the conclusions drawn adequately supported by the results?

Yes

Are sufficient details of methods and analysis provided to allow replication by others?

Yes

Reviewer Expertise:

NA

I confirm that I have read this submission and believe that I have an appropriate level of expertise to confirm that it is of an acceptable scientific standard.

# Response

**Published:** 2020-01-23

**Authors:** Gholib Gholib

**Affiliations:** Universitas Syiah Kuala, Indonesia

Response to Reviewer 1.

First, we would like to thanks to the reviewer 1 for approved our paper. Her comments and suggestions have helped us to further improve the paper. As you will see in the response-to-reviewer  below, we have answered all points raised and have changed the paper accordingly. 

1. Do not use personal pronouns. Please don't use we, I etc******

***Response: It has been changed *throughout the text *.***

2. Choose the most 5 important words

 Response: ***In the guideline, it was mentioned that we can use until 8 keywords.We still  keep the keywords because using more keywords will help people to find our paper.***

3. Do not use abbreviation word on the beginning of the line

***Response: It has been changed throughout the text.***

4. Is there any correlation between C and T in this case?

Response: ***Yes there is a correlation between cortisol and testosterone in some animals from previous studies.* From the previous study, high levels of cortisol can suppres the testosterone production.**

***An additional sentence and references have been added in the revised paper***

5. Due to the same base form of steroid (cyclopentanoperhydrophenantrend), we can use the same kit for animal as in human.

Response: ***Thank you for your suggestion, This statement has been mentioned in the discussion section.***

6. Do not use personal pronouns

Response: ***It has been changed throughout texts***

 7. Should be done by veterinarians

Response: ***During the study, our team was accompanied by a veterinarian during data collection.Blood samples were performed by veterinarians. This statement has been written in the paper ( ethical statement, collection and processing of blood samples)***

8. How to validate the data, because the samples are less than it should be?

Response: ***We have added the number of samples. Now, 32 Aceh cattle were used: 16 bulls and 16 cows. *For the *testosterone validation test, all cattle ( 16 bulls and 16 cows) were used. For cortisol validation test,  16 cattle ( 8 bulls and 8 cows ) were used which is taken from 32 Aceh cattle above.  The explanation  has been added in the revised paper***

*9. Please add some information of the feed and the volume per head.*

*Response:* **Feed given to the cattle is grass at 10% of body weight and 1% of body weight of rice bran and tofu dregs. The explanation has been added in the revised paper**

*10. until the assay process are conducted*

***Response: Thank you for your suggestion, we have added some words in revised paper***

*11. Please add sign of significant on testosterone*

***Response: Superskrip “ a” has been added to the testosterone data in the Figure 3, because they showed not significant different. * Table 2. has been deleted from the revised paper.**

*12. Due to the same base form, the kit also can be used in animals as in human. Please add this explanation*

*Response:* **The explanation has been added in the discussion section, paragraph 3 ^rd^ in revised paper**

*13. Please explain about gradient slope that is used in the research as standard. Is there prerequisite to determine parallelism standart?*

*Response:* ***To determine whether curve of serial dilution samples was parallel with the curve of hormone standard (C and T), the slope of the serial dilution samples curve must be compare with the slope of the standard curve. For this, we can do a statistical analysis to compare the value of the slope from dilution samples curve and standard curev. We can determine that the both slopes (sample curve and standard curve) were parallel when there was not significant different between slope of dilution sample curve with standard curve. From our results, Slope of Sample one and sample two were not significant different with the slope of the standard curve (p>0.05). It is indicated that both slope are similar.***

***14.*** *Why biology validation should be done? Is parallelism test based on dilution can be confirmed? In the real condition, does the treatment should be validated by biology validation? Does parallelism test not enough?*

**Response: To test the reliability of the aasay, there are several validations should be done namely: analytical validation, physiological validation, and biological validation. Parallellism is one part of the analytical validation. This test aimed to evaluate whether antigen (hormone) can bind with the antibody in the assay accurately and find the optimal dilution if needed.** **However, parallelism  test is not a marker of specificity of the assay, but a**

**proof of a dose–response relationship (Möstl *et al*., 2005).** **Therefore, it is not possible to select**

**the best-suited assays to assess physiological stress and reproduction only based on the parallelism results. To achieve that, it is important to perform a biological validation or physiological validation. For physiological validation of stress hormone,  a widely used method is an adrenocorticotrophic hormone (ACTH) challenge test by injecting animals with ACTH (for review see Tauma & Palme 2005 ) and by injecting radiolabeled testosterone [4(C)-14C testosterone] for  testosterone validation (Möhle et al. 2002).**
